# EA Ameliorated Depressive Behaviors in CUMS Rats and Was Related to Its Suppressing Autophagy in the Hippocampus

**DOI:** 10.1155/2020/8860968

**Published:** 2020-09-22

**Authors:** Zhinan Zhang, Xiaowen Cai, Zengyu Yao, Feng Wen, Zhiyi Fu, Jiping Zhang, Zheng Zhong, Yong Huang, Shanshan Qu

**Affiliations:** ^1^School of Traditional Chinese Medicine, Southern Medical University, Guangzhou, Guangdong Province, 510515, China; ^2^Nanfang Hospital, Southern Medical University, Guangzhou, Guangdong Province, 510515, China

## Abstract

Autophagy is confirmed to be involved in the onset and development of depression, and some antidepressants took effect by influencing the autophagic process. Electroacupuncture (EA), as a common complementary treatment for depression, may share the mechanism of influencing autophagy in the hippocampus like antidepressants. To investigate that, sixty Sprague-Dawley rats firstly went through chronic unpredictable mild stress (CUMS) model establishment, and 15 rats were assigned to a control group. After modeling, 45 successfully CUMS-induced rats were randomly divided to 3 groups: CUMS, selective serotonin reuptake inhibitor (SSRI), and EA groups (15 rats per group), to accept different interventions for 2 weeks. A sucrose preference test (SPT), weighing, and open field test (OFT) were measurement for depressive behaviors of rats. Transmission electron microscope (TEM), immunohistochemistry (IHC), and western blot analysis were used to evaluate the autophagic changes. After that, depression-like behaviors were successfully induced in CUMS models and reversed by SSRI and EA treatments (both *p* < 0.05), but these two therapies had nonsignificant difference between each other (*p* > 0.05). Autolysosomes observed through TEM in the CUMS group were more than that in the control group. Their number and size in the SSRI and EA groups also decreased significantly. From IHC, the CUMS group showed enhanced positive expression of both Beclin1 and LC3 in CA1 after modeling (*p* < 0.05), and the LC3 level declined after EA treatments, which was verified by decreased LC3-II/LC3-I in western blot analysis. We speculated that CUMS-induced depression-like behavior was interacted with an autophagy process in the hippocampus, and EA demonstrated antidepressant effects by partly inhibiting autophagy with a decreased number of autolysosomes and level of LC3 along with LC3-II/LC3-I.

## 1. Introduction

Depression is a common mental disorder that severely limits psychosocial functioning and diminishes quality of life [1], affecting over 300 million people worldwide [2]. It contributes the most global all years lived with disability ranked by the WHO as well as the main reason blamed for suicide [2]. Many molecular mechanisms are involved in the etiology of the disorder, among which autophagy has been put forward as one of them [3].

Autophagy is a widely existed protein degradation method in eukaryotic cells, which is usually induced and upregulated by external stimuli. Three forms of autophagy are mainly described: macroautophagy, microautophagy, and chaperone-mediated autophagy, among which the macroautophagy is the most common type. In macroautophagy, expendable cytoplasmic constituents are targeted and isolated from the rest of the cell in the form of autophagosomes. After that, autolysosomes fused with available lysosomes and autophagosomes are degraded and recycled.

A variety of proteins are involved in the regulation of autophagy, among which the Beclin1 and LC3 are the typical ones. Beclin1, a homologous of yeast ATG6, is a regulatory gene of autophagy. Besides, the modification process of LC3, microtubule-associated protein 1 light chain 3, is very important for the formation of autophagosomes. The precursor of LC3 was processed into LC3-I by cysteine protease Atg4B. After the catalyzing of Atg7, intracytoplasmic LC3-I transformed into a form of membrane bound, namely, LC3-II [4]. The number of autophagosomes is associated with the ratio of LC3-II/LC3-I. Therefore, Beclin1 and LC3 are important markers when autophagy occurs.

Autophagy is in fact a conserved lysosomal degradation pathway essential for the central nervous system. It plays an important role in neuronal development and synaptic plasticity. The dysfunction of autophagic degradation leading to accumulation of abnormal protein inside the neuronal cells is the common basis of neurodegenerative diseases, such as Parkinson's disease, Alzheimer's disease, and Huntington's disease [5]. There is increasing evidence showing that stressors can induce autophagy [6]. Exposure to various kinds of environmental stressors is a convincing cause to depression, by which a depression animal model, chronic unpredictable mild stress (CUMS) model, has been established and widely used [7]. Some recent researches showed autophagy alteration in CUMS animals with the reversal of behavioral effect [8, 9].

Autophagy has been frequently discussed in the researches of depression about human and animal. A small sample-size study found higher expression of autophagy genes in blood mononuclear cells from depression patients than that from healthy controls [10], and another study presented the positive correlation between the expression of Beclin1 in these cells and clinical treatment success [11]. Since the hippocampus is one of the key brain areas where depression develops, autophagy is often discovered in this location. Liu et al. observed the autophagy activation of the hippocampus in the depression model of rats, such as increased autophagosomes, LC3-II/LC3-I ratio, and Beclin1 level in neurons and the atrophied brain area [12]. Hence, autophagy was inhibited in the hippocampus of OBX rats (a depression animal model) and upregulated by fluoxetine (a widely used antidepressant belonging to selective serotonin reuptake inhibitors (SSRIs)) with reversal of depressive-like behavior and enhanced expression of LC3-II, Beclin1, etc. [13]. Other studies also presented decreased autophagic markers [14, 15].

Acupuncture and electroacupuncture (EA) are effective complementary therapies for depression based on antidepressant treatment [16]. The effect on autophagy of acupuncture or electroacupuncture (EA) has been proven in several diseases. Tian et al. found that acupuncture could clear *α*-synuclein in the substantia nigra par compacta of the brain in a PD mouse model [17]. With decreased levels of LC3 and Beclin1, EA may alleviate the cerebral ischemia/reperfusion by inhibiting neurons' excessive autophagy [18]. Meanwhile, autophagy may be beneficial that the LC3 expression of autophagy had positive correlation with neurologic function in a hemorrhagic stroke rat model [19].

However, although the investigation of autophagy in depression is comparatively thorough and a certain number of studies about acupuncture and autophagy have published, the comprehensive research discussing the acupuncture impact on autophagy in depression is still limited. Therefore, this experiment was designed to investigate the autophagy phenomenon in hippocampus neurons of CUMS rats and attempt to find out the antidepression mechanism of EA underneath.

## 2. Materials and Methods

### 2.1. Animals

Seventy-five male Sprague-Dawley rats weighing 180-220 g, provided by Southern Medical University Experimental Animal Center (Guangdong, China; license No. SYXK (Yue) 2016-0167), were housed individually in the SPF facility (temperature 24 ± 2°C, humidity 50-60%) at Southern Medical University, China. After 3-day adaption, CUMS models began to be established in all rats except for 15 rats in the control group. The study protocol was approved by Southern Medical University Experimental Animal Ethics Committee (NO. L2017178) and followed the United States National Institutes of Health Guide for the Care and Use of Laboratory Animals (NIH Publication No. 85-23, revised 1986).

### 2.2. Chronic Unpredictable Mild Stress (CUMS) Model Establishment

The CUMS model was established referring to previous studies [20–23] that evaluate biological effects of antidepressants. Except for the control group, the remaining 60 rats underwent a 21-day CUMS procedure modified on Zhang et al. [24], during which the rats were exposed to different stressors including water deprivation (24 h), food deprivation (24 h), immobilization (2 h), level shaking (5 min), and tail clamping (3 min; 3 cm from the end of the tail). These stressors were processed randomly as one stressor per day on rats, and the same stressor was not applied consecutively over two days to avoid animals' prediction of the occurrence of stimulation (Table 1). According to our former experiment, about 75~80% CUMS rats could be successfully induced, which was similar to the literature [25], and these CUMS-induced rats were then randomly and equally assigned to the CUMS, SSRI, and groups, followed by respective interventions. Besides, the control group received normal breeding.

### 2.3. Intervention

The whole intervention lasted for 14 days after CUMS modeling [24]. After CUMS modeling, rats in the EA group underwent EA at GV20 (at the midpoint between the auricular apices) and GV29 (at the midpoint between the medial ends of the two eyebrows) [26]. Disposable acupuncture needles (0.30 mm × 25 mm, Hwato Appliance Factory) were inserted in both acupoints horizontally to 5 mm depth. Following the insertions, the needles were connected to electrodes for electrical simulation with sparse waves (1 mA in electric current, 2 Hz in frequency, and 5 V in voltage). The stimulus intensity was preferable when the rat's head slightly trembled. The whole EA treatment was implemented for 30 min each time, once per day. Rats in the SSRI group were given paroxetine (1.8 mg/kg/d, i.g.) [27] after 30-minute gentle immobilization. The same volume of saline (per kg as paroxetine) was also applied on the EA group. For the control and CUMS groups, only gentle immobilization and saline administration were used once per day during the same period.

### 2.4. Behavioral Analysis

Three parameters including sucrose preference test (SPT), weighing, and open field test (OFT) were examined at the end of intervention to evaluate the depression-like behavior of rats. The rats were scheduled for euthanasia the day after OFT.

#### 2.4.1. Sucrose Preference Test (SPT) and Weighing

The procedure of SPT was performed as described previously [20, 28]. Firstly, rats were trained to adapt 1% sucrose solution (weight in volume (*w*/*v*)) in their home cages with two bottles of 1% sucrose solution placed in each cage. Twenty-four hours later, 1% sucrose in one bottle was replaced with tap water and continued adapting for 24 h. After adaptation, rats were deprived of water and food for 24 h, followed by rats weighing. During the 1 h SPT, rats were housed in individual cages and had free access to two bottles containing 200 ml of sucrose solution (1% *w*/*v*) and 200 ml of water, respectively. Each bottle was weighed before and after the test, and two bottles were changed randomly to prevent place preference. The sucrose preference was calculated as the percentage of the consumed 1% sucrose solution relative to the total amount of liquid intake. At the end of the test, all animals were returned to their home group housing with normal breeding.

#### 2.4.2. Open Field Test (OFT)

The OFT is often employed to evaluate the effects of antidepressant treatment in animals [29]. In this study, it was used to measure exploratory behavior and general activity in rats, performed as previous research [20]. The apparatus was a 100 cm × 100 cm × 38 cm black wooden box which was kept in an isolated room with normal lighting and temperature. The floor of the arena was divided into 25 cm × 10 cm × 10 cm squares. A video recording system was stationed above the apparatus to capture the movement of rats within the box. Subsequently, each rat was placed in the center of the open field without any agitation, and their explorative movement was measured for 5 min using the video recorder. After one test of a rat, the apparatus was cleaned to abolish the odor of the former tested rat. A neutral observer stayed away from the apparatus during the test. When OFT was finished, the video was analyzed using SMART 3.0, and the time in the center area and total distance traveled were assessed.

### 2.5. Transmission Electron Microscope (TEM)

Eight rats from each group were anesthetized with 10% chloral hydrate anesthesia (3 ml/kg, intraperitoneal injection), followed by perfusion with a mixture of 2.5% glutaraldehyde and 2.5% paraformaldehyde. Then, the left CA1 of each hippocampal tissue was separated immediately, and immersion fixation was completed at around 1 mm^3^ size. Samples were rinsed in cold phosphate-buffered saline (PBS, 4x for 15 min) and placed in 2.5% glutaraldehyde until the operation of TEM. First, they were immersed in 1% osmium for 1 hour and rinsed in PBS (3x for 15 min). Next, they were immersed in ascending concentrations of acetone (50, 70, and 90%, each for 15 min; 100%, 3x for 15 min). After being immersed in mixed liquor of acetone and Spurr's resin (acetone : Spurr′s resin = 1 : 1 for 1 h; acetone : Spurr′s resin = 1 : 2 for 2 h), they were quickly immersed in Spurr's resin at room temperature overnight and then embedded in coffin molds in Spurr's resin, curing for 8 h at 70°C in an oven (Shanghai Yiheng, DHG-9053A). 60-nm-thick ultrathin sections were cut (Leica, UC7) and counterstained with saturated aqueous uranyl acetate and Reynolds' lead citrate (3x for 5 min). Sections were photographed with TEM (Hitachi, H-7500) at 10,000x and 40,000x magnifications.

### 2.6. Immunohistochemistry (IHC)

For IHC, the left CA1 region of random 24 rats from each group was fixed with 4% paraformaldehyde, embedded in paraffin, and sectioned. After being rehydrated, the samples were immersed with 3% H_2_O_2_ at room temperature for 10 min to block endogenous horseradish peroxidase (HRP) activity, and antigen retrieval was performed by microwave for 8 min in citrate buffer. Each section was incubated with normal goat serum in PBS for 30 min at room temperature and then incubated with primary antibody (1 : 100; anti-Beclin1 antibody, Abcam; anti-LC3 antibody, Abcam) at 4°C overnight. After phosphate-buffered saline (PBS) washing, the slides were then incubated with a corresponding second antibody (Abcam) at room temperature for 30 min and stained with diaminobenzidine (DAB; ChemMate TM DAKO Envision TM Detection Kit, DAKO). The slides were then counterstained with hematoxylin, dehydrated, and mounted. The degree of staining was controlled by microscopic observation (Olympus) with 200x magnification. The IHC scores were calculated by the software ImageJ which showed percent contribution of different grades of positive.

### 2.7. Western Blot Analysis

The left CA1 tissues of random 16 rats from each group were homogenized in lysis buffer, and the lysis process was continued for 30 min at 4°C. After being centrifuged at 12000 rpm for 15 min, the protein contents were determined by bicinchoninic acid protein assay (Beyotime Institute of Biotechnology, Shanghai, China). 20 *μ*g of proteins of each sample was loaded into wells of 12% SDS-PAGE gel, electrophoretically separated, and transferred on to PVDF membrane. After being blocked in 5% skim milk/TBST at room temperature for 3 h, the membranes were incubated with specific primary antibodies including anti-Beclin1 (1 : 1000; Abcam), anti-LC3 (1 : 2000; Abcam), and anti-GAPDH (1 : 2000; Proteintech) in incubation boxes at 4°C for 16 h. The membranes were washed with TBST for 3 times with 5 min each before and after the incubation of secondary antibody [1 : 2000; HRP-conjugated Affinipure Goat Anti-Rabbit IgG (H+L)]. Finally, images were acquired using darkroom development techniques for chemiluminescence (ProteinSimple, FluorChem E, USA).

### 2.8. Statistical Analysis

All data were presented as mean ± SD both complied with normal distribution. One-way ANOVA followed by LSD post hoc tests was used to evaluate differences for behavioral analysis, IHC, and TEM results among groups. A *p* value < 0.05 was considered to be statistically significant. All calculations were made using SPSS 22.0 and GraphPad Prism 8.0 (GraphPad Software, Inc., San Diego, CA, USA).

## 3. Results

### 3.1. Behavior Analysis

**Figure 1:**
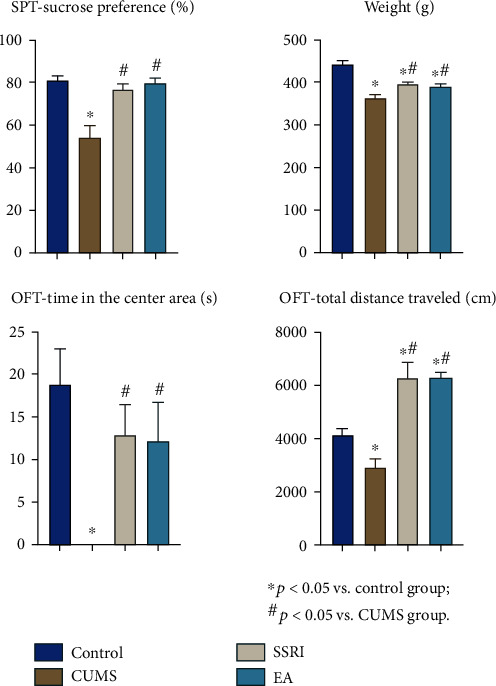
Comparison of SPT, weight, and OFT among 4 groups (mean ± SEM) (the OFT-time in the center area of the CUMS group was too much shorter than those of the other groups to show in the bar graph, but the specific number is listed in Table 2) (*n* = 15).

Firstly, excluding the modeling failure, there were 45 remaining rats presented CUMS-induced depressive behaviors according to evaluation of SPT, weight, and OFT, with a successful modeling rate of about 75%. They were then immediately divided into the CUMS, SSRI, and EA groups (15 per group) and accepted different treatments. CUMS models were stable in the course of treatment with significantly different behavioral results compared with the control group (*p* < 0.05). No rats died during the model establishment or intervention period. Secondly, the SSRI and EA groups also showed positive results compared with the CUMS group, which indicated the improvement of depression-like behavior of rats after both treatments (*p* < 0.05), but they were nonsignificantly different between each other (*p* > 0.05) (Table 2 and Figure 1).

Difference among groups was analyzed with one-way ANOVA as normal distribution complied, and LSD was used for a post hoc test with homogeneity of variance satisfied. ^∗^*p* < 0.05 vs. the control group; ^#^*p* < 0.05 vs. the CUMS group.

### 3.2. TEM

**Figure 2:**
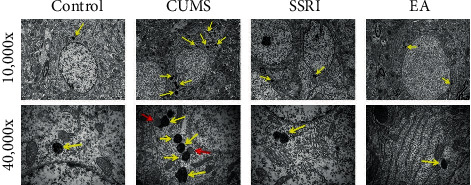
Number and size of autolysosomes in hippocampus CA1 neurons in each group (yellow arrows, autolysosomes; red arrows, autophagosomes; magnification, 10,000× (2 *μ*m) and 40,000× (500 nm)) (*n* = 2).

Autolysosomes (vacuum-like bilayer structures enveloping the cell contents) of five rats from each group were observed through TEM and highlighted by yellow arrows in Figure 2. The size and number both increased in the CUMS group than those in the control group, which proved the activation of autophagy after modeling. Compared with those in the CUMS group, the number and size of autolysosomes in the SSRI and EA groups decreased significantly, but it was hard to distinguish the difference among them from Figure 2.

### 3.3. IHC

**Figure 3:**
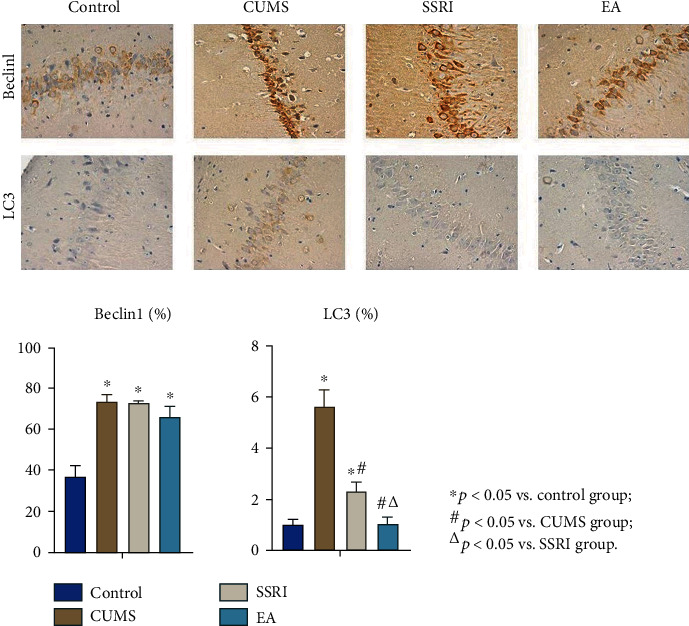
Expression of Beclin1 and LC3 in hippocampus CA1 of each group (IHC, 200x magnification; mean ± SEM; *n* = 6).

With ImageJ, we chose the sum of percent contribution of positive to compare the expression intensity of two targets in CA1. The difference of Beclin1 and LC3 among groups was significant (*p* < 0.05). During the post hoc test, the CUMS group showed significant higher positive expression of Beclin1 and LC3 than the control group, indicating the activation of autophagy in CA1 after modeling (*p* < 0.05). The Beclin1 level in the SSRI or EA group was higher compared with that in the control group, respectively (*p* < 0.05), but there was no significant difference between these two groups (*p* > 0.05). Moreover, the LC3 level declined after SSRI and EA treatments, and the effect of EA in regulating LC3 expression was greater than that of SSRI (*p* < 0.05) (Table 3 and Figure 3).

Difference among groups was analyzed with one-way ANOVA as normal distribution complied, and LSD was used for the post hoc test with homogeneity of variance satisfied. ^∗^*p* < 0.05 vs. the control group; ^#^*p* < 0.05 vs. the CUMS group; ^△^*p* < 0.05 vs. the SSRI group.

### 3.4. Western Blot Analysis

**Figure 4:**
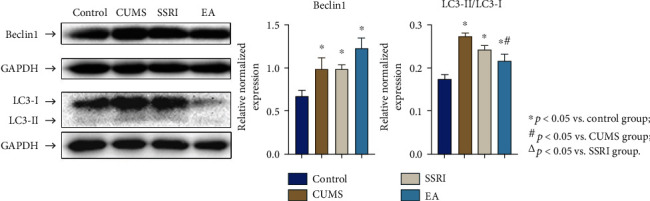
Expression of Beclin1 and LC3-II/LC3-I in hippocampus CA1 of each group (relative normalized; mean ± SEM; *n* = 4).

The difference of relative normalized Beclin1 and LC3-II/LC3-I expression among groups was significant (*p* < 0.05). During the post hoc test, Beclin1 level trend was in accordance with IHC results. The CUMS, SSRI, and EA groups all showed significant higher positive expression of Beclin1 than the control group (*p* < 0.05), but they did not significantly distinguish with each two of them (*p* > 0.05). As for LC3-II/LC3-I, this ratio was significantly higher in the CUMS, SSRI, and EA groups compared with the control group, respectively. LC3-II/LC3-I also declined after EA treatments (*p* < 0.05), which agreed with IHC results. Moreover, the difference between the SSRI and CUMS or EA groups did not reach statistical significance (*p* > 0.05) (Figure 4).

## 4. Discussion

As a widely applied animal model for depression, the CUMS model presents depressive behaviors like anhedonia and decreased locomotor activity. Anhedonia is the core symptom of depression with the characteristic as lacking enjoyment of food in human or animal models. Loss of weight may then result from the reduced food intake. SPT is recognized as a common method to evaluate the anhedonia in animals, and the reduced preference for sucrose in the test is a key indicator of depression in rodents [28]. Besides, the OFT measured locomotor activity, with reduced locomotor activity indicating anxiety-like behaviors associated with depression [30]. Hence, we used SPT, weighing, and OFT in the evaluation of depressive behaviors of CUMS rats in this study. After modeling, the CUMS rats displayed conspicuous depressive-like symptoms such as anhedonia, decline in spontaneous locomotor functions, and weight loss. The results were consistent with findings in previous reports [31], supporting the success of the modeling.

From the results of TEM in our experiment, autolysosomes in the hippocampus neurons observed in CUMS rats were more than those in the control ones. What is more, EA or SSRI also reduced the number and size of autolysosomes compared with the CUMS group. Considering all the results above, SSRI and EA may improve depressive behaviors through impacting the autophagy level of hippocampus neurons. Although there are limited depression researches learning the relationship between autophagy and EA, the correlation of autophagy in the hippocampus and depression has attracted growing attention. Autophagosomes, the form before autolysosomes, were reported to increase in the hippocampus of chronic restraint stress-exposed rats, another widely used depression animal model [32]. Notably, autophagosomes could also exert a protective effect in alleviating hippocampus neuronal apoptosis along with amelioration of depressive-like behaviors [33]. So, the specific mechanism of autolysosomes changes induced by EA in the hippocampus of CUMS rats still waits for further investigation.

Autophagy is essential for basal homeostasis. Beclin1 is a key regulator of autophagy in mammalian cells [34], and LC3 is a reliable marker of autophagosomes [35]. Both expression levels can reflect the autophagy activity of cells. To firstly provide the direct evidence linking the interaction between EA and autophagy to depression, we assessed the expression of autophagic biomarkers, including Beclin1 and LC3, in the CA1 of rats' hippocampus following CUMS.

Given by our study, CUMS rats presented depressive behaviors and enhanced expression of Beclin1 and LC3 according to IHC. When Beclin1 is activated, a lot of membrane sources (the production center of autophagosomes) are formed in the cytoplasm. In the process from phagophore to autolysosomes, LC3-I transferred from the cytoplasm to LC3-II on the membrane of autophagosomes. LC3 functions in autophagy substrate selection and autophagosome biogenesis [36]. Its membrane-located form LC3-II has a positive correlation with the amount of autophagosomes [37], and it is degraded by lysosomal enzyme after autophagosomes fusing with lysosomes into autolysosomes [38]. The higher expression of Beclin1 and LC3 in the CUMS group suggested the activation of autophagy in this depression animal model. In addition, the ratio of LC3-II/LC3-I that closely related to the number of autophagosomes could be tested as additional research to verify the changes of LC3 [39]. In the western blot analysis, the ratio decreased in the EA group after treatment but not in the SSRI group. Hence, both positive results from IHC and western blot analysis supported the effect of EA on LC3 expression in CUMS models.

Actually, CUMS can either activate the pathway involving Beclin1 and LC3 in hippocampus [40] or inhibit it [14, 15, 41]. In addition, these two markers can also be differently influenced in other models or types of depression. For example, Beclin1 and LC3 were upregulated in electroconvulsive shock-induced depressive rats [42] and downregulated in pain- or LPS-induced depression rats as well as maternal separation rats which were accompanied with depressive behaviors [43–45]. The function of autophagy in the nervous system is still controversial. In the circumstances, the effect of autophagy on depression may be bilateral. For example, hydrogen sulfide, an antidepressant-like compound for diabetic rats, could improve the depression-like behavior of rats by enhancing hippocampal autophagy through BDNF-TrkB pathway [46], but the similar improvement was explained by opposite mechanism in another research, in which the upregulating BDNF-TrkB pathway was along with the decreasing autophagy in hippocampus [47]. Maybe the effect of autophagy on depression varies from different types or treatments.

One of the major brain areas where EA ameliorates depressive behaviors of animals is the hippocampus, in which several mechanisms have been confirmed, such as synaptic plasticity, neuroinflammation, and neurotransmitter upregulation [48–50]. However, whether and how autophagy participates in the regulation of EA on depression are unclear yet. Furthermore, apoptosis is closely relevant to autophagy as they, respectively, constitute distinct mechanisms for the turnover or destruction of cytoplasmic structures within cells and of cells within organisms. An apoptosis-related research discovered that acupuncture could improve depressive behaviors of psychological stress-induced depression rats by suppressing oxidative stress-mitochondrial apoptotic pathway in the hippocampus [51]. It is likely that EA could affect autophagy in the hippocampus of depression models.

Treatments that rescued behavioral deficits of CUMS animals by autophagy were mostly based on the Belin1 and/or LC3 pathway in the hippocampus, accompanied with the changes of autophagosomes or autolysosomes. Animal studies have showed the activation of Beclin1 and LC3-II following fluoxetine treatment in the hippocampus, and this alteration could be observed in microglia [40, 52]. Additionally, some other brain regions were involved in the regulation of autophagy in CUMS animal models, such as the prefrontal cortex, in which andrographolide may produce antidepressant-like effects in CUMS-induced mice by upregulating autophagy [53]. Collecting the evidence above, these mentioned therapies may reverse inhibited autophagy in the hippocampus or other brain areas under CUMS-induced depression state.

EA has been proven to interact with critical autophagic markers or autolysosomes. Although the influence of autophagy in depression is waiting to be verified, EA has shown neural protection against or with autophagy in other neural degenerated diseases. The regulation of acupuncture or EA on autophagy has been mainly discussed in cerebral ischemia reperfusion (CIR). Autophagy is a crucial part in the impairment of CIR and could be improved or suppressed by EA dependent on time in CIR animal models [18, 54, 55], indicating that EA may play a dual role in CIR autophagy. Besides, in central poststroke pain rat models, EA could relieve symptoms by inhibiting autophagy in the hippocampus [56], which could enlighten the study of EA on autophagy in depression.

In this present study, SSRI and EA did not yet influence Beclin1 increase triggered by CUMS, but reduced the level of LC3 in the hippocampus after modeling where EA showed advantage over SSRI. As mentioned above, LC3 is closely relative to the number of autolysosomes; thus, the autolysosome decline from TEM was connected with the lower LC3 level of the EA group than the SSRI or CUMS group. The results of TEM and IHC suggested that EA probably participated in the formation of autolysosomes related with LC3. Furthermore, both Beclin1 and LC3 are involved in the regulation of autophagy intensity and duration [18]. Collecting the different variations of Beclin1 and LC3, we considered that EA took effect on relieving depressive behaviors through inhibition of LC3-involved autolysosomes formation, but not Beclin1.

Although this article is a preliminary observation about EA's influence on the autophagy process in the hippocampus of CUMS rats, many remarkable characteristics of acupuncture therapy have been explained from the perspective of autophagy mechanism. For example, acupuncture induces an increase in autophagy in the brain by inserting needles into the legs and avoids the difficulty of crossing the blood-brain barrier for conventional drugs [55], which provides a new idea for green medicine.

There are still some limitations in the study. Based on the inconsistent autophagic changes in the hippocampus of CUMS animal models and the close relationship with apoptosis, the neural apoptosis could be added to verify the effect of autophagy. This is a primary research about EA's effect on autophagy in depression models; hence, research about investigation of apoptosis and the specificity of EA with sham-acupoint control is considerable in the future.

## 5. Conclusion

Although the interrelationship among EA, autophagy, and depression is interesting and blank, our data preliminarily provided the evidence that the occurrence of CUMS-induced depression-like behavior may be concerned with autophagy, and EA demonstrated antidepressant effects by partly inhibiting autophagy with the decreased level of LC3 and number of autolysosomes. This study raised the possibility that EA ameliorated depressive behaviors in CUMS rats by suppressing the autophagic level in the hippocampus.

## Figures and Tables

**Table 1:** The stressors of the 21-day CUMS procedure.

Stressor	Duration
Water deprivation	24 h
Food deprivation	24 h
Immobilization	6 h
Level shaking	5 min
Tail clamping	3 min

**Table 2:** Comparison of SPT, weight, and OFT among 4 groups (mean ± SD).

Group	SPT-sucrose preference (%)	Weight (g)	OFT-time in the center area (s)	OFT-total distance traveled (cm)
Control (*n* = 15)	80.87 ± 8.53	441.02 ± 38.19	18.72 ± 16.43	4124.67 ± 941.30
CUMS (*n* = 15)	54.05 ± 22.35^∗^	363.18 ± 28.32^∗^	0.04 ± 0.10^∗^	2925.52 ± 1197.65^∗^
SSRI (*n* = 15)	77.15 ± 7.94^#^	394.44 ± 24.40^∗,#^	12.87 ± 13.80^#^	6267.50 ± 2387.70^∗,#^
EA (*n* = 15)	79.54 ± 9.08^#^	388.99 ± 31.24^∗,#^	12.17 ± 17.61^#^	6287.90 ± 755.56^∗,#^
*F*	16.455	4.795	19.310	13.400
*p*	<0.05	0.05	<0.05	<0.05

**Table 3:** Positive expression of Beclin1 and LC3 (mean ± SD, percent).

Group	Beclin1	LC3
Control (*n* = 6)	37.04 ± 12.87	1.02 ± 0.46
CUMS (*n* = 6)	73.92 ± 7.63^∗^	5.61 ± 1.63^∗^
SSRI (*n* = 6)	73.27 ± 1.27^∗^	2.33 ± 0.90^∗,#^
EA (*n* = 6)	66.40 ± 11.71^∗^	1.05 ± 0.61^#,△^
*F*	20.081	27.757
*p*	<0.05	<0.05

## Data Availability

All data that support the findings are included in the article.
